# Fe_3_O_4_–Au cluster–satellite particles as direct colorimetric reporters in a smartphone-quantified lateral flow immunoassay for prostate-specific antigen

**DOI:** 10.1007/s00604-026-08422-8

**Published:** 2026-09-25

**Authors:** Daniele Marra, Erica Cavaliere, Daniela Terracciano, Luca Livio Levorato, Tina D’Aponte, Vincenzo Iannotti, Francesco Gentile, Raffaele Velotta, Bartolomeo Della Ventura

**Affiliations:** 1 Department of Physics “Ettore Pancini”, University of Naples Federico II, Via Cintia 26, Naples, 80126 Italy; 2 Department of Translational Medical Sciences, University of Naples Federico II, Via S.Pansini, 5, Naples, 80131 Italy; 3 Department of Experimental and Clinical Medicine, University Magna Græcia, Campus Universitario Salvatore Venuta, Viale Europa, Catanzaro, 88100 Italy

**Keywords:** Lateral flow assay, Prostate-specific antigen, Fe₃O₄–Au cluster–satellite magnetic particles, Gold nanoparticles, Colorimetric detection, Smartphone-based detection, Point-of-care testing, Immunoassay

## Abstract

Fe₃O₄–Au cluster–satellite magnetic particles (CSMPs) were investigated as high-payload direct colorimetric reporters in a smartphone-quantified sandwich LFA for prostate-specific antigen (PSA) determination in undiluted human serum. Commercially available PEGylated dextran-coated Fe₃O₄ magnetic clusters were used as scaffolds, decorated with multiple gold nanoparticles, and subsequently functionalized with anti-PSA antibodies through photochemical immobilization. Magnetic recoverability facilitated particle isolation during synthesis and bioconjugation, whereas assay transduction remained exclusively optical, without magnetic actuation, external excitation, or post-assay signal amplification. Systematic variation of reporter extinction revealed a non-monotonic relationship between particle loading and analytical performance. Low particle loading produced weak optical signals, whereas excessive loading caused early saturation and compression of the test-to-control response. An intermediate extinction provided the best balance between signal intensity, low-concentration detectability, and usable dynamic response. Under optimized conditions, the assay provided an estimated limit of detection of 0.25 ng·mL⁻¹ within 20 min. The assay showed limited interference from human kallikrein 2, intra- and inter-assay coefficients of variation below 10%, and preliminary agreement with a reference chemiluminescent immunoassay in seven human serum samples. These results demonstrate that CSMPs can serve as direct high-contrast colorimetric reporters for quantitative LFA while preserving a simple smartphone-based readout.

**Graphical abstract:**

**Figure:**
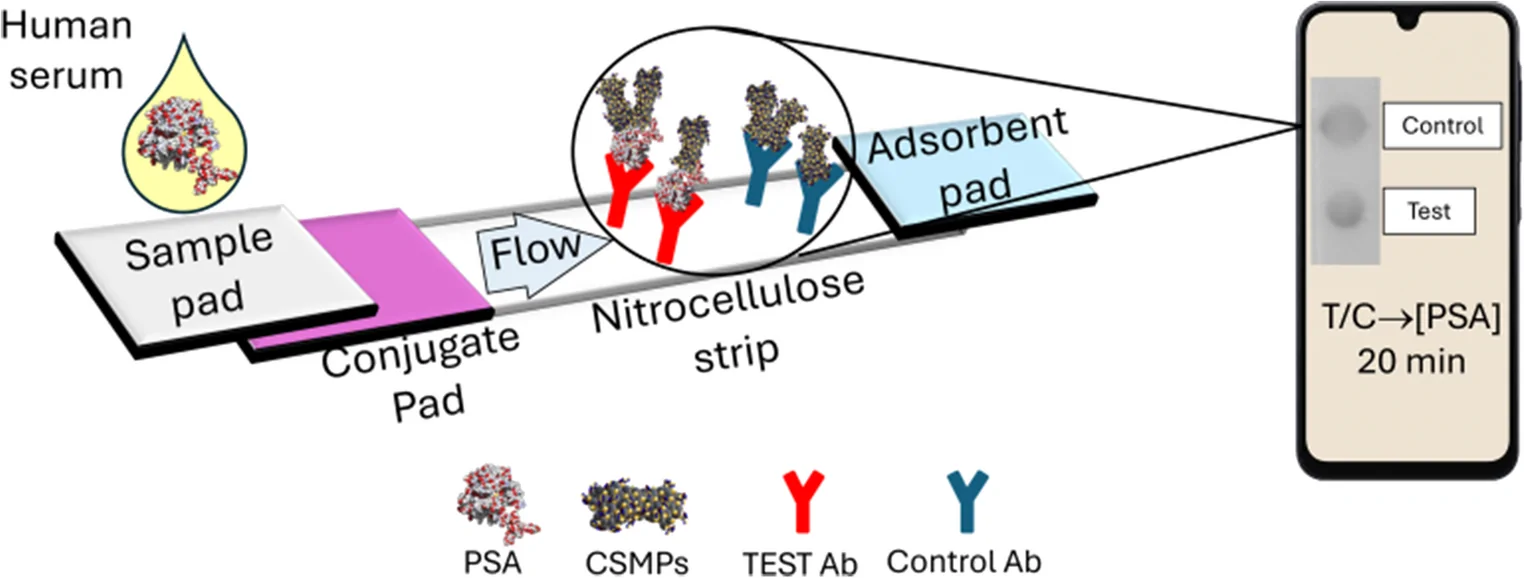

**Supplementary Information:**

The online version contains supplementary material available at https://doi.org/10.1007/s00604-026-08422-8.

## Introduction

Lateral flow assays (LFA) are among the most established point-of-care (POC) analytical platforms because they combine rapid capillary-driven operation, low manufacturing cost, portability, and visual interpretation [1, 2]. In conventional colorimetric formats, gold nanoparticles (AuNPs) are the most widely used signal reporters owing to their intense localized surface plasmon resonance, chemical stability, and compatibility with different bioconjugation strategies [3, 4]. Despite these advantages, the transition from qualitative detection to reliable quantitative analysis remains challenging. At low analyte concentrations, the number of nanoparticles captured at the test zone may be insufficient to generate adequate optical contrast, particularly in complex biological matrices. This limitation is partly associated with the finite optical extinction and biomolecular payload of individual AuNPs reporters and ultimately restricts the analytical sensitivity and quantitative resolution achievable through direct colorimetric detection [5, 6].

A wide range of signal-amplification and alternative-transduction strategies has therefore been developed to improve LFA performance. Fluorescent lanthanide nanoparticle reporters have enabled high-sensitivity quantitative LFA detection [7], while persistent luminescent nanophosphors have been employed for multiplexed analyte detection [8]. Surface-enhanced Raman scattering has been integrated with LFA for sensitive prostate specific antigen (PSA) quantification [9], whereas electrochemical transduction has been coupled to the strip format to provide quantitative PSA readout [10]. Magnetic sensing and magnetic-field-assisted approaches represent another class of LFA enhancement strategies [11], while Fe_3_O_4_-Au supraparticles have been exploited as photothermal reporters [12]. Direct colorimetric detection can also be intensified through post-assay metal deposition or enzymatic amplification [6]. Although these strategies can substantially decrease detection limits, they frequently require additional incubation steps, external excitation sources, dedicated detectors, or more complex signal-processing procedures. Consequently, sensitivity enhancement may transform an intrinsically simple test strip into an instrument-dependent or multistep analytical system. The development of reporters capable of generating stronger optical signals while preserving the operational simplicity of conventional colorimetric LFA therefore remains an important objective in POC biosensor design. This trade-off is particularly relevant to point-of-care PSA testing, where the development of quantitative methods must balance analytical performance with affordability, accessibility, and operational simplicity, while optical quantification often relies on dedicated readers [13]. Improving the intrinsic optical response of the reporter without adding assay steps or specialized instrumentation therefore represents an attractive strategy for PSA-LFA development.

Multifunctional reporters with a high optical payload provide a promising route to address this challenge. Core–satellite particles consist of a large carrier decorated with multiple AuNPs, thereby concentrating numerous optically active domains and biomolecular recognition sites within a single transported and captured object [14]. Their potential for colorimetric LFA has been demonstrated using silica-supported plasmonic structures. In particular, Au–Ag assemblies formed on silica particles enabled visual semiquantitative detection of PSA, illustrating how multiple metallic domains supported on a larger scaffold can generate intense optical contrast [15]. Gold nanoshell reporters have also been shown to improve PSA-LFA performance relative to conventional spherical AuNPs under directly comparable assay conditions, supporting the use of plasmonic labels as an alternative to post-assay amplification [16]. However, the conventional preparation of silica-based plasmonic reporters commonly involves several processing stages, including silica synthesis, surface silanization, preparation and immobilization of metallic seeds, metal overgrowth, and repeated centrifugation and washing steps [17].

Magnetoplasmonic core–satellite particles represent an attractive alternative that combines multiple plasmonic domains with magnetic recoverability [18, 19]. Magnetic nanoparticles have been investigated in LFA both as directly visible labels and as functional components for magnetic separation, enrichment, focusing, or quantitative detection [11, 20]. More complex Fe_3_O_4_-Au particles have also been employed as multifunctional reporters in SERS- and photothermal-based LFA, achieving high analytical sensitivity through Raman enhancement or photothermal conversion [12, 21]. In these systems, however, the magnetic or hybrid nature of the reporter is generally coupled with additional actuation, excitation, or detection instrumentation. Their use as colorimetric reporters in quantitative LFA, without magnetic manipulation during assay operation and without excitation-based or spectroscopic detection, remains comparatively underexplored.

In the present work, commercially available polymer-functionalized Fe_3_O_4_ clusters were used as scaffolds to assemble multiple AuNPs satellites, following a cluster–satellite particle structure previously developed and structurally characterized by our group [22]. The resulting cluster–satellite magnetic particles (CSMPs) provide an AuNP-rich surface for antibody immobilization and concentrate multiple plasmonic domains within a single reporter. Their magnetic properties facilitate selective separation during synthesis and bioconjugation, whereas their optical extinction enables direct colorimetric transduction in the sandwich LFA. We therefore hypothesized that CSMPs could operate as high-payload colorimetric reporters in LFA while retaining efficient release from the conjugate pad, capillary transport through nitrocellulose, and quantitative signal generation in complex biological samples.

PSA was selected as a clinically relevant analyte to evaluate this concept. PSA testing is an important component of prostate cancer early-detection pathways, risk assessment, and clinical management. However, PSA is not cancer-specific, and its concentration should be interpreted in conjunction with clinical findings, individual risk factors, and imaging results [23]. From an analytical perspective, PSA represents a challenging target for colorimetric LFA, because clinically relevant concentrations are in the low ng·mL⁻¹ range. Moreover, measurement in undiluted serum requires adequate colloidal stability, minimal nonspecific retention on the membrane, and sufficient analytical specificity to distinguish PSA from structurally related kallikreins, particularly human kallikrein 2 (hK2) [24].

**Fig. 1:**
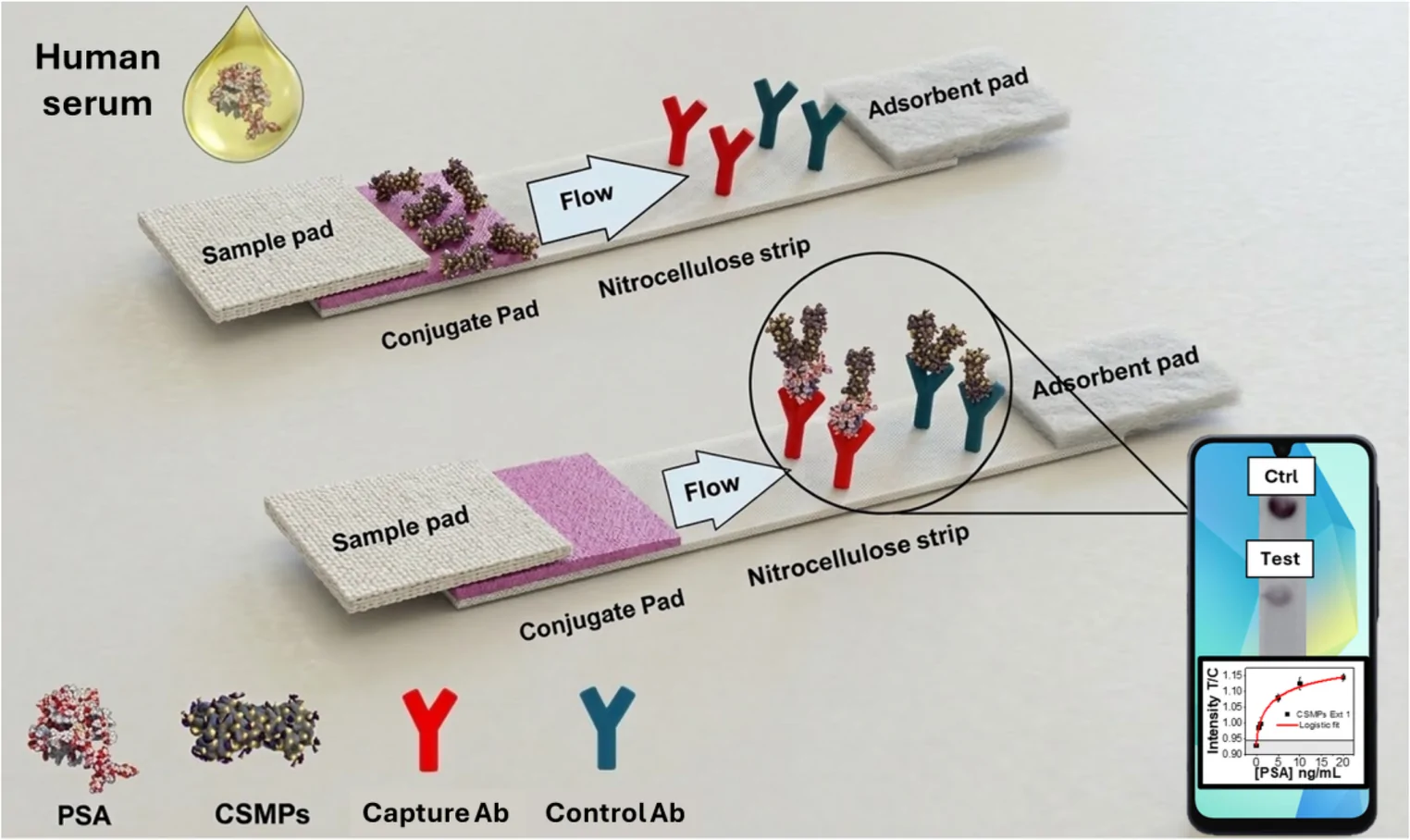
Operating principle of the CSMP-based LFA for PSA detection. CSMPs bind the target analyte when present in the sample and migrate along the strip by capillary action. At the test zone, an immobilized anti-PSA antibody captures the analyte–particle complexes, generating a visible colorimetric signal. The particles that are not retained at the test zone continue to migrate towards the control zone, where they are captured by control antibodies, confirming proper assay performance. After 20 min, the strip image is acquired using a smartphone, and the signal is quantified by image analysis, enabling the determination of PSA concentration in the sample

Here, we demonstrate the integration of CSMPs as direct colorimetric reporters in a quantitative LFA. By combining the optical features of CSMPs with smartphone-based image analysis and photochemical immobilization technique-mediated antibody functionalization, the proposed platform (Fig. 1) enables sensitive PSA quantification in human serum without signal amplification or dedicated optical instrumentation, achieving a limit of detection below 1 ng·mL^-1^ within 20 min. Furthermore, the high optical contrast of CSMPs enables naked-eye detection, highlighting their potential for future integration into simple and low-cost home diagnostic systems.

## Experimental

### Instrumentation and materials

Milli-Q ultrapure water (resistivity 18.5 MΩ·cm) was used for the preparation of all solutions. Tetrachloroauric acid trihydrate (HAuCl_4_·3H_2_O), sodium citrate (Na_3_C_6_H_5_O_7_), polysorbate 20 (Tween-20, C_26_H_50_O_10_), sucrose (C_12_H_22_O_11_), phosphate-buffered saline (PBS, pH 7.4), lyophilized bovine serum albumin fraction V was purchased from Microtech (Italy).

PEGylated dextran-coated Fe_3_O_4_ magnetic composite particles (MCPs) (nanomag-D, product code: 09-55-252) were obtained from MicroMod (Rostock, Germany). These particles exhibit a core–shell-like architecture consisting of a magnetite core formed by aggregates of iron oxide nanocrystals and a dextran shell, with PEG-NH_2_ surface functionalization.

Anti-Prostate-Specific Antigen antibodies [EPR22271-97] (capture) and [EPR22271-81] (detector), and Anti-Rabbit IgG Fc antibody [RMG02], human prostate-specific antigen from human semen (P3338-25UG), recombinant human kallikrein 2/Kl (AMab229375-10ug) were purchased from Abcam company. Human serum samples from healthy female donors and PSA-positive male participants were provided by the Azienda Ospedaliera Universitaria Federico II (Naples, Italy). Samples were collected in accordance with the Declaration of Helsinki under approval no. 180/20 of the Research Ethics Committee of the AOU Federico II, University of Naples Federico II. All participants provided written informed consent before enrollment, and the samples were anonymized prior to analysis.

Nitrocellulose membranes (Hi-Flo^™^ Plus 75), glass fiber conjugate pads, absorbent pads, sample pads were obtained from Merck Millipore (Billerica, MA, USA).

The instrumentation used for CSMPs synthesis and optical characterization included a mechanical motor stirrer (Heidolph RZR 50, Germany), a hot plate (DLAB MS-H28-PRO), a spectrophotometer (Jenway 6715 UV/VIS), and an ultrasonic bath operating at 37 kHz (Elmasonic S 40 (H)).

Magnetic separation was performed using an axially magnetized NdFeB disk magnet (supermagnete.com) with dimensions of 30 mm in diameter and 10 mm in thickness. Hydrodynamic size and ζ -potential measurements were carried out using a Zetasizer Nano ZS (Malvern Instruments). Morphological characterization was performed by Transmission Electron Microscopy (TEM) using a JEOL JEM-2100 microscope (JEOL Ltd., Japan). Magnetic measurements were performed using a vibrating-sample magnetometer (DSM 880, Microsense).

### CSMPs synthesis

CSMPs were synthesized according to a well-established protocol developed by our group with minor modifications [22]. Briefly, MCPs (136 µL, 10 mg·mL^-1^) were dispersed in 100 mL of Milli-Q water and heated to boiling under vigorous mechanical stirring (900 rpm). Once boiling was reached, HAuCl_4_·3H_2_O solution (400 µL, 10 mg·mL^-1^) and Na_3_C_6_H_5_O_7_ (6.65 mL, 10 mg·mL^-1^) were sequentially added to the reaction mixture. Under these conditions, citrate ions acted simultaneously as reducing and stabilizing agents, promoting the reduction of Au^3+^ and the nucleation of gold onto the surface of the magnetic cores while providing electrostatic stabilization. The reaction was maintained for 15 min under continuous stirring, during which the solution colour gradually changed from brown to burgundy, indicating the formation of gold-coated magnetic nanoparticles. The reaction was stopped by rapidly cooling the suspension in an ice bath. The resulting CSMPs were purified by magnetic separation using a magnet placed under the beaker for 2 h to remove unreacted reagents and free gold nanoparticles. The particles were then resuspended in Milli-Q water and sonicated for 10 min to reduce aggregation prior to further characterization.

### CSMPs functionalization

Surface functionalization of the CSMPs was carried out using rabbit monoclonal anti-PSA antibodies (detector) through the photochemical immobilization technique (PIT) [25]. This approach relies on the selective photoreduction of disulfide bonds within the IgG structure, generating reactive thiol groups that can form stable covalent interactions with thiol-reactive gold surfaces, without the need for additional chemical reagents. This immobilisation mechanism promotes a preferential orientation of the antigen-binding domains toward the surrounding medium, resulting in enhanced biosensing performance. The robustness of PIT has also been independently validated in a comparative study focused on LFA, where it was identified among the most effective strategies for antibody immobilisation on gold substrates [26].

The functionalization protocol began with the UV activation of the antibody solution. 500 µL of anti-PSA antibody (10 µg·mL^-1^) was placed in a quartz cuvette and irradiated for 30 s using a Trylight UV source equipped with two U-shaped low-pressure mercury lamps (6 W each, emission at 254 nm) (Figure S1a). The lamps were positioned symmetrically around the cuvette to ensure a uniform irradiation intensity of approximately 0.3 W·cm^-2^. After the irradiation, the activated antibody solution and the CSMPs suspension were transferred into separate test tubes and simultaneously injected into the fluidic setup illustrated in Figure S1b. Two silicone tubes delivered the respective solutions into a 3D-printed spiral static micromixer [27]. The functionalized particles (f-CSMPs) were directly collected at the mixer outlet.

A blocking step was performed using 1 mL of 1 mg·mL^-1^ BSA solution, corresponding to the total volume obtained after mixing 500 µL of antibody solution with 500 µL of CSMPs, which is compatible with the proposed 1:1 mixing system. Excess unreacted antibodies and BSA were removed by magnetic separation for 1 h. Successful surface functionalization and blocking were confirmed by UV–Vis spectroscopy, which showed a plasmonic red shift of approximately 3 nm (551 to 554 nm) after functionalization, reflecting an increase in the local refractive index around the AuNPs upon antibody immobilization. No additional shift was observed following the BSA blocking step, suggesting that all available surface sites had already been saturated (Figure S2).

After removal of the unbound fraction, the f-CSMPs suspension was diluted with Milli-Q water to reach the desired optical extinction and sonicated for 3 min to break up possible aggregates, making the particles ready for subsequent biosensing experiments. The functionalization parameters described above were optimized according to the procedure established in our previous work [28].

### Lateral flow strip assembly

The LFA strips were assembled from four functional components: a nitrocellulose (NC) membrane, a sample pad, a conjugate pad, and an absorbent pad. Each element was prepared individually prior to the final strip assembly. The NC membrane was cut to a width of 0.5 cm using a guillotine cutter. The sample pad, made of glass fibre, was cut to dimensions of 1 × 2 cm and pretreated by immersion in an aqueous solution containing 0.05% Tween-20 under gentle rocking for 1 h, followed by overnight drying at 50 °C.

The conjugate pad, also composed of glass fibre, was cut to dimensions of 1 × 0.5 cm and treated under the same agitation and drying conditions using a blocking solution containing 1% BSA, 5% sucrose, and 0.5% Tween-20. After treatment, 5 µL of f-CSMPs solution at the required extinction value were deposited onto the conjugate pad and allowed to dry at room temperature for 2 h.

The absorbent pad (0.5 × 1 cm) required no chemical pretreatment. The treated components were then assembled according to the configuration shown in Fig. 1.

Functionalization of the NC membrane was carried out by depositing two spots of capture reagents. The test dot was formed by spotting 0.5 µL of anti-PSA antibody solution (capture) (1 mg·mL^-1^), while the control dot was made by spotting 0.5 µL of anti-rabbit antibody solution (1 mg·mL^-1^) at 0.5 cm from the test. The membranes were incubated at 37 °C for 2 h to allow antibody adsorption.

Blocking was then performed by immersing the strips in a solution containing 1% BSA in PBS under gentle rocking for 5 min, followed by a washing step in PBS supplemented with 0.05% Tween-20 for 1 min. Finally, the membranes were stored overnight at 4 °C prior to use.

### Sensor operating principle

The sensor operates according to a sandwich-format LFA. Briefly, 180 µL of undiluted human serum is applied onto the sample pad and migrates along the strip by capillary flow. The sample first reaches the conjugate pad, where f-CSMPs are pre-loaded. During this stage, and as it migrates along the nitrocellulose membrane, the f-CSMPs interact with the target analyte, leading to the formation of PSA–CSMPs immunocomplexes. Upon reaching the test zone, the complexes are captured by immobilized monoclonal anti-PSA antibodies through specific recognition of PSA, forming a sandwich immunocomplex that generates a visible signal. Excess f-CSMPs, including particles that did not bind PSA or were not retained at the test zone, continue migrating toward the control zone. Here, immobilized anti-rabbit monoclonal antibodies recognize the rabbit anti-PSA antibodies conjugated to the CSMPs, forming immunocomplexes that produce a visible control signal, thereby confirming the correct operation of the assay. The simultaneous appearance of both test and control signals indicates a positive result. The presence of only the control dot indicates PSA concentrations below the sensor visual LoD, whereas the appearance of the test in the absence of the control dot indicates an invalid test. After 20 min from sample deposition, the signal can be quantified by acquiring an image of the strip using a smartphone. The image is then processed in ImageJ, and the test-to-control intensity ratio (T/C) is correlated with the PSA concentration.

### Image processing and data analysis

Images of the LFA strips were acquired using an iPhone 15 under standardized conditions. To ensure reproducible imaging geometry, all photographs were captured using a custom 3D-printed smartphone holder (Figure S3), which maintained a fixed distance and position between the smartphone and the test strip throughout all acquisitions. The images were converted to 8-bit grayscale and analyzed using ImageJ. Prior to signal quantification, image intensity was calibrated using a grayscale calibration card (Figure S4a). The grayscale reference was analyzed by measuring the intensity of each grayscale level using the *Measure* function in ImageJ. The resulting intensity values were normalized, assigning a value of 1 to the darkest level (black) and 0 to the brightest level (white). A calibration curve was then generated from the normalized grayscale values (Figure S4b) and applied to all subsequent image analyses using the Rodbard calibration function in ImageJ. For each strip, regions of interest (ROI) corresponding to the test and control spots were manually defined using the polygon selection tool, and the mean grayscale intensities were extracted. Each experimental condition was analyzed in triplicate. For each experimental condition, the test (T) to control signal (C) intensity ratio was calculated for each replicate and selected as sensing parameter. The obtained T/C values were then averaged within each condition to generate a mean T/C value, and the corresponding standard deviation was calculated. Calculation of T/C for each individual strip preserves the paired nature of the test and control measurements and intrinsically accounts for their covariance before averaging across replicates. Consequently, common-mode fluctuations affecting both signals in the same direction can be partially normalized in the resulting ratio.

## Results and discussion

### CSMPs characterization

**Fig. 2:**
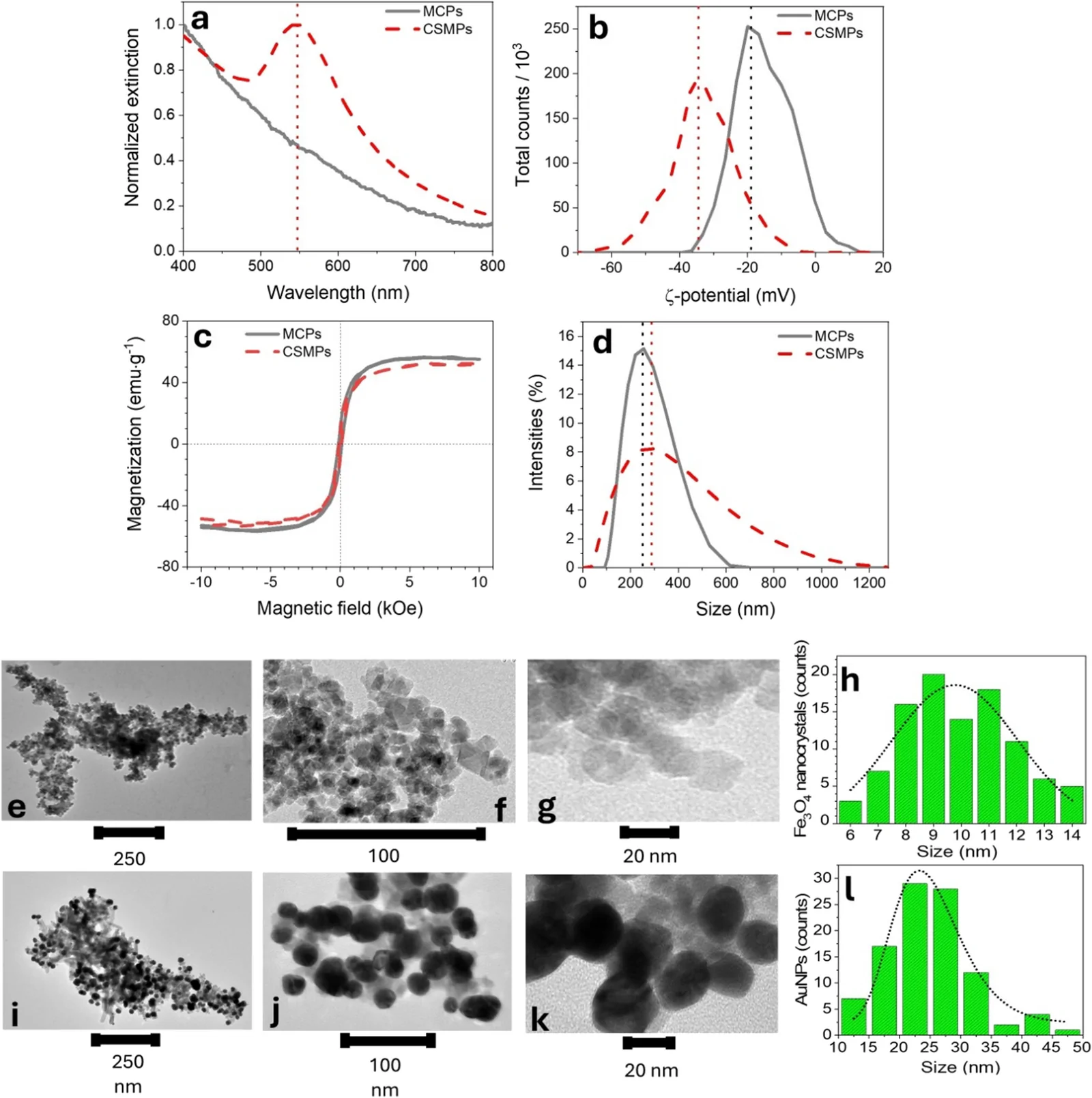
Characterization of magnetic clusters (MCPs) and cluster–satellite magnetic particles (CSMPs). (**a**) Normalized extinction spectra. (**b**) ζ-potential distributions. (**c**) Magnetic hysteresis loops. (**d**) Hydrodynamic size distributions determined by dynamic light scattering (DLS). (**e**–**h**) TEM micrographs of MCPs at different magnifications and the corresponding magnetite nanocrystal size distribution histograms. (i–l) TEM micrographs of CSMPs at different magnifications and the corresponding AuNPs size distribution histograms

All the characterizations of the CSMPs are depicted in Fig. 2. The extinction spectra of the particles, normalized to their maximum values, are presented in panel (a). MCPs alone exhibit an extinction profile without defined peaks (grey solid line), whereas the CSMPs display a pronounced plasmonic resonance centred at approximately 550 nm (red dashed line), confirming the formation of gold nanoparticles on the magnetic cores. Surface charge characterization by ζ-potential measurements (b) provided further insight into the colloidal stability of the two systems. Bare MCPs dispersed in water exhibited a ζ-potential peak around − 18 mV, whereas after gold coating, the CSMPs displayed a more negative ζ-potential of approximately − 35 mV, indicative of enhanced electrostatic stabilization [29].

Magnetic characterization by hysteresis loop measurements at 300 K (c) yielded saturation magnetization values of 55 emu⋅g^-1^ for MCPs and 51 emu⋅g^-1^ for CSMPs. The modest decrease in magnetization following gold coating reflects the diamagnetic contribution of gold.

The hydrodynamic size distributions determined by DLS (d) showed a peak centred at approximately 250 nm for MCPs and a broader distribution centred at approximately 280 nm for CSMPs, consistent with the deposition of AuNPs on the magnetic clusters.

Morphological characterization was performed by TEM. Representative micrographs of MCPs and CSMPs at different magnifications are shown in Fig. 2e–l, together with the corresponding size distribution histograms of the magnetite nanocrystals and AuNPs. TEM analysis revealed an average diameter of 10 ± 3 nm for the Fe_3_O_4_ nanocrystals constituting the magnetic clusters, while the AuNPs decorating the CSMPs exhibited an average diameter of 24 ± 7 nm. A comprehensive structural and compositional characterization of these CSMPs, including high-resolution electron microscopy and elemental mapping analyses confirming the cluster–satellite architecture, has been previously reported by our group [28].

### Optimization of CSMP reporter loading and analytical performance

**Fig. 3:**
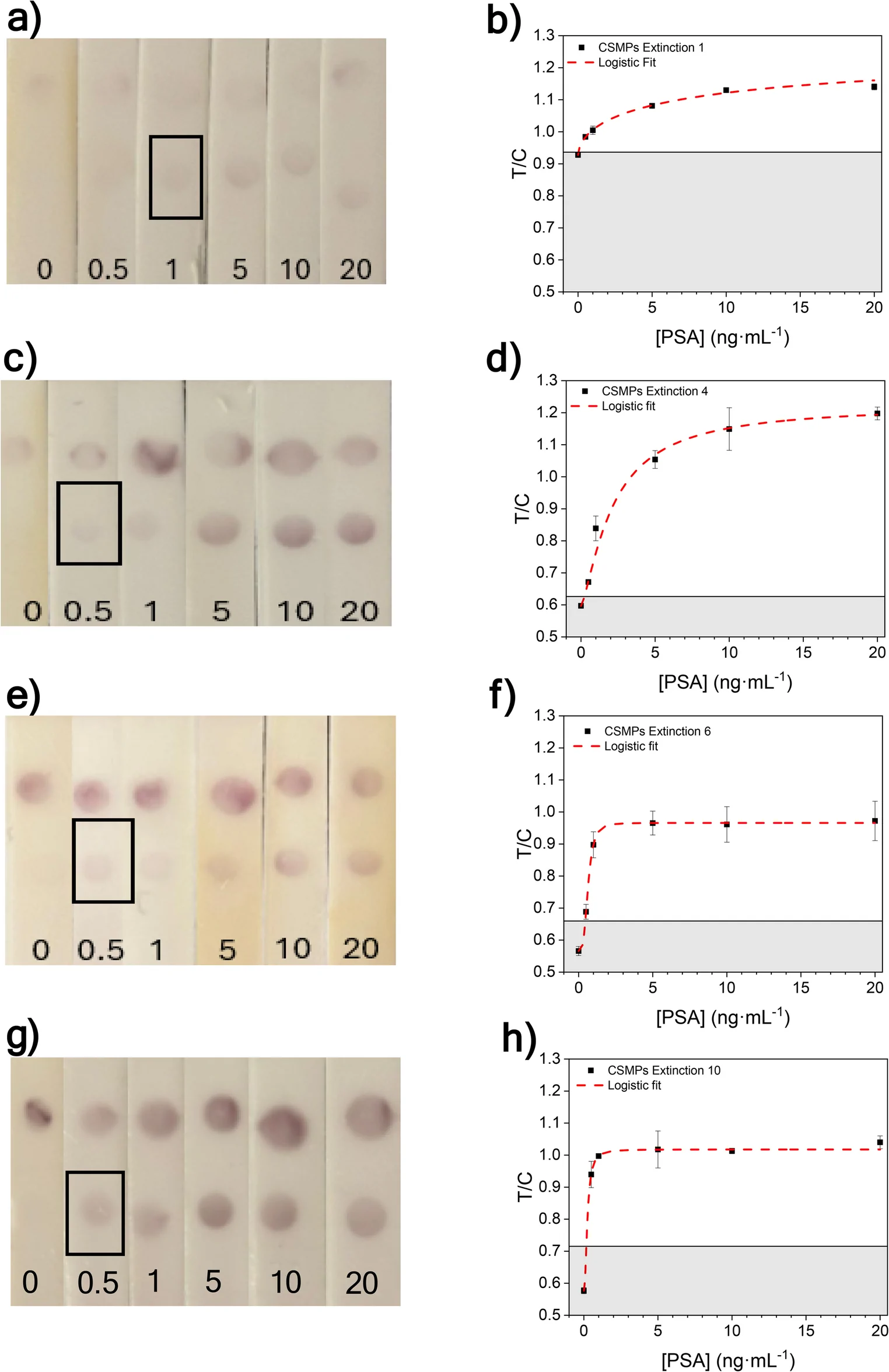
Effect of CSMP reporter loading on assay performance. Representative lateral flow strips obtained using functionalized CSMP suspensions with optical extinction values of 1 (**a**), 4 (**c**), 6 (**e**), and 10 (**g**), for PSA concentrations ranging from 0 to 20 ng·mL⁻¹ in spiked human serum. Corresponding dose–response curves obtained by smartphone image analysis using the test-to-control signal ratio are shown in panels (**b**), (**d**), (**f**), and (**h**), respectively. The grey areas represent the background region defined as the mean blank response plus three standard deviations. Black rectangles indicate the lowest PSA concentration producing a visually discernible test signal. Data are reported as mean ± standard deviation of triplicate measurements. Ext 4 provided the best balance between detection capability and quantitative response and was selected for subsequent experiments

A key step in assay development was the optimization of CSMP reporter loading to identify the condition providing the best combination of detection capability and quantitative response. Functionalized CSMP suspensions with optical extinction values of 1, 4, 6, and 10 were evaluated using female donor serum lacking endogenous PSA and spiked with PSA concentrations ranging from 0 to 20 ng·mL⁻¹. Each condition was tested in triplicate, and strip images were acquired 20 min after sample application. The assay response was quantified by smartphone image analysis using the test-to-control signal ratio (T/C). Representative strips and the corresponding dose–response curves are shown in Fig. 3.

For comparative assessment of the different reporter-loading conditions, the background region was defined as the mean blank response plus three standard deviations and is represented by the grey area in the dose–response plots. The corresponding LoD was estimated by inversion of the fitted calibration curve. The black rectangles in Fig. 3 indicate the lowest PSA concentration producing a visually discernible test signal. Because the LoD estimates were obtained from triplicate measurements, they were primarily used to compare the different reporter-loading conditions rather than as a formal validation of the assay detection limit.

At the lowest reporter loading, Ext 1, both test and control signals were weak. The blank response was already close to the lower portion of the concentration-dependent signal range. Combined with the small standard deviation obtained from the triplicate blank measurements, this elevated baseline placed the mean blank + 3SD threshold close to the blank response, and inversion of the fitted curve returned an estimated LoD of 0.02 ng·mL⁻¹. However, this value fell below the experimentally investigated concentration range and was associated with a weak absolute signal and a narrow usable response window. Consistently, a clearly discernible test signal was observed only from 1 ng·mL⁻¹ PSA.

Increasing reporter loading to Ext 4 markedly enhanced the optical contrast of both test and control zones. A visually discernible test signal was obtained at 0.5 ng·mL⁻¹, while inversion of the fitted calibration curve yielded an estimated LoD of 0.25 ng·mL⁻¹. More importantly, this condition produced the broadest and most progressive concentration-dependent variation of the T/C ratio, allowing improved discrimination among PSA concentrations throughout the investigated range.

The individual test- and control-zone signals underlying the Ext 4 response are reported in Figure S5. Both profiles show irregular point-to-point variations superimposed on their overall concentration-dependent trends, whereas these irregular variations are markedly attenuated in the corresponding T/C curve shown in Fig. 3d. Because T and C were measured on the same strip and their ratio was calculated separately for each replicate, common-mode fluctuations affecting both detection zones can be partially compensated. In particular, positively correlated variations can reduce the propagated variance of the ratio through the covariance term, contributing to a smoother and more robust quantitative response without completely correcting zone-specific variability.

Further increases in reporter loading to Ext 6 and Ext 10 generated stronger absolute signals but caused the T/C response to approach a plateau at relatively low PSA concentrations. This early saturation compressed the usable response range and reduced quantitative discrimination, despite estimated LoDs of approximately 0.5 and 0.2 ng·mL⁻¹ for Ext 6 and Ext 10, respectively. In particular, the relatively low numerical LoD obtained at Ext 10 was accompanied by a markedly restricted concentration-dependent response and therefore did not translate into improved overall quantitative performance.

This behavior can be attributed to two concurrent effects. First, the capture antibodies immobilized at the test line represent a finite binding capacity, which is approached at progressively lower PSA concentrations as reporter extinction increases, since more PSA is already captured by CSMPs during strip migration. Second, as already noted, given the enhanced plasmonic properties of the reporters, each CSMP carries multiple AuNPs, its optical extinction markedly exceeds that of a single AuNP reporter, so that even a modest number of captured particles generates a locally high optical density, driving the signal toward the upper limit. Together, these effects compress the concentration-dependent T/C response at relatively low PSA concentrations, despite the higher absolute signals observed at Ext 6 and Ext 10. Overall, Ext 4 provided the best combination of low-concentration detectability, optical contrast, and usable dynamic response. This reporter loading was therefore selected for all subsequent analytical characterization and human-serum measurements.

Direct colorimetric PSA lateral-flow assays based on conventional gold reporters have been investigated for more than two decades. An early colloidal-gold immunostrip enabled the detection of free and total PSA in serum with a visual detection threshold of approximately 1 ng·mL⁻¹ within 20 min and was subsequently evaluated in 51 male serum samples, although quantitative analysis required densitometric readout and the assay included a washing step [30]. More recently, a quantitative AuNP-based LFIA for total PSA achieved a detection limit of 0.3 ng·mL⁻¹ in spiked serum using photoscanner-based signal quantification [3]. These studies show that conventional AuNP reporters can already provide sub-ng to low-ng mL⁻¹ PSA detection, but also illustrate the continuing trade-off between analytical performance and simplicity of quantitative readout.

An alternative strategy is to increase the intrinsic optical response of the reporter itself. In a directly comparative PSA LFA, gold nanoshell reporters produced higher colorimetric signals and a fivefold lower detection limit than conventional 40 nm gold nanospheres [16]. The Fe₃O₄–Au CSMPs investigated here exploit the same general high-optical-payload rationale by carrying multiple AuNP satellites on each magnetic cluster, thereby increasing the optical contribution associated with each captured reporter relative to a single conventional AuNP label. Under optimized conditions, the assay provided an estimated LoD of 0.25 ng·mL⁻¹, comparable to the lower values reported for conventional AuNP-based PSA LFAs, while enabling smartphone-based image acquisition and analysis of undiluted human serum samples.

### Specificity, recovery and real sample evaluation

To evaluate the analytical selectivity of the proposed CSMP-based LFA, an interference assay was performed using hK2, a serine protease sharing high structural and sequence homology with PSA [24]. PSA samples at 0.5, 5, and 20 ng·mL⁻¹ were supplemented with hK2 at a tenfold higher mass concentration, corresponding to 5, 50, and 200 ng·mL⁻¹ hK2, respectively. For each PSA concentration, the result obtained in the presence of hK2 was normalized to that of the corresponding PSA-only sample, which was set to 100%.

**Fig. 4:**
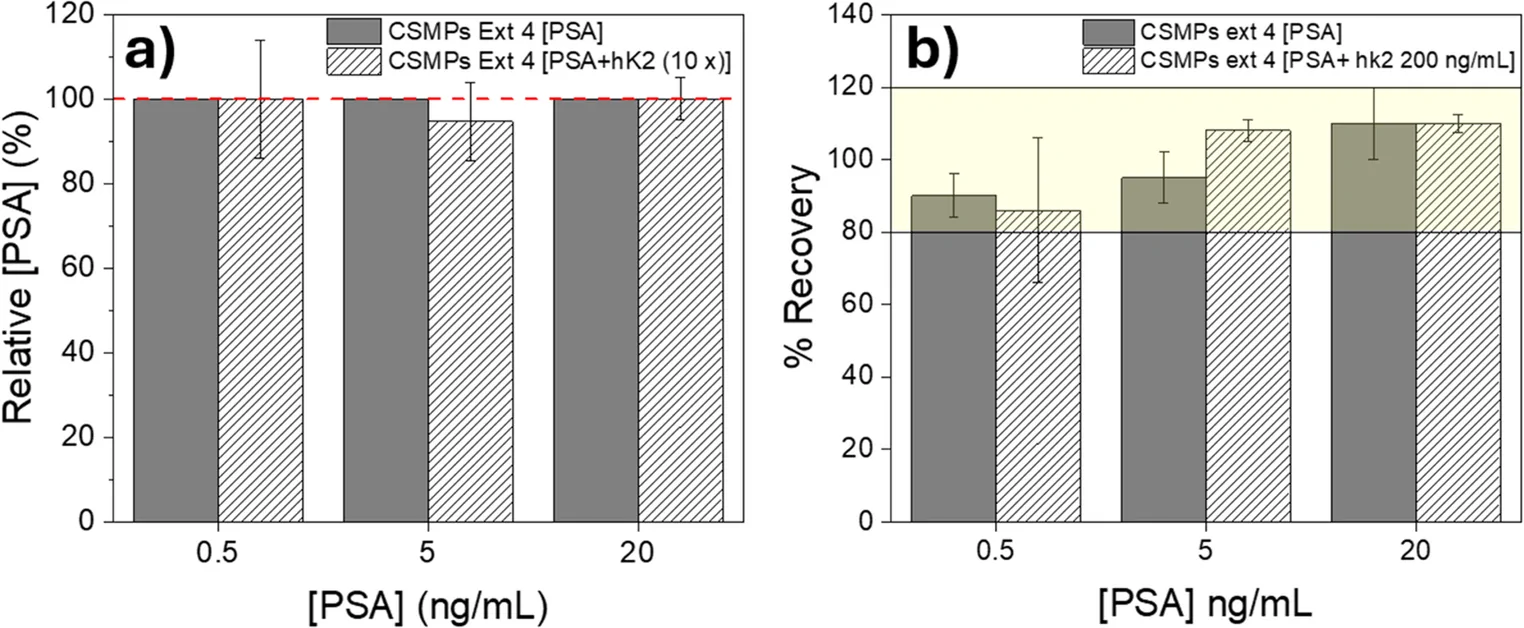
Assessment of hK2 interference and PSA recovery. (**a**) Specificity assessment of the CSMPs-based lateral flow assay toward PSA in the presence of hK2. PSA samples at 0.5, 5, and 20 ng·mL⁻¹ were supplemented with hK2 at a 10:1 hK2-to-PSA mass-concentration ratio, corresponding to hK2 concentrations of 5, 50, and 200 ng·mL⁻¹, respectively. The measured PSA concentrations were normalized to the corresponding PSA-only samples (red dashed line). (**b**) Recovery of PSA measured using the CSMPs-based lateral flow assay in the absence and presence of 200 ng·mL^-1^ hK2. Recovery was calculated as (measured concentration/expected concentration) × 100 for PSA concentrations of 0.5, 5, and 20 ng·mL^-1^. The yellow shaded area indicates the predefined recovery acceptance interval (80–120%)

As shown in Fig. 4a, the CSMP-based LFA response remained close to that obtained for the corresponding PSA-only samples when hK2 was present at a 10:1 hK2-to-PSA mass-concentration ratio, with relative deviations below 5% across the investigated concentration range. These results indicate limited interference from hK2 under the tested conditions.

To further assess the quantitative reliability of the assay, recovery experiments were performed by analyzing PSA samples at the same concentrations used before in the absence and presence of 200 ng·mL^-1^ hK2. Recovery was calculated as the ratio between the measured and expected PSA concentrations. As shown in Fig. 4b, all recovery values fell within, or very close to, the predefined acceptance interval (80–120%), confirming that acceptable PSA quantification was maintained despite the presence of hK2. Specifically, the assay yielded recoveries of 90%, 95%, and 110% in the absence of hK2, and 86%, 108%, and 110% following hK2 addition. The absence of any systematic bias toward lower or higher recoveries further demonstrates that hK2 does not significantly interfere with PSA determination under the investigated experimental conditions.

The assay showed good repeatability and between-run precision at the three investigated PSA concentrations. Intra-assay CVs were calculated from triplicate measurements performed within the same analytical run, whereas inter-assay CVs were calculated across three independent assay runs, each performed in triplicate. All CV values were ≤ 9% and therefore below the predefined acceptance threshold of 20% (Table 1).

**Table 1:** Intra-assay repeatability and inter-assay precision of the CSMP-based LFA at Ext 4, evaluated at PSA concentrations of 0.5, 5, and 20 ng·mL⁻¹. Intra-assay CVs were calculated from triplicate measurements within the same analytical run, whereas inter-assay CVs were calculated across three independent assay runs, each performed in triplicate

[PSA] (ng·mL^− 1^)	Intra-assay CV (%)	Inter-assay CV (%)
0.5	8	6
5	2	8
20	3	9

To evaluate the applicability of the proposed immunoassay to clinically relevant samples, 7 human male serum samples were analysed without dilution, with each sample measured in triplicate. The PSA concentrations determined by the proposed assay were compared with those obtained at the Azienda Ospedaliera Universitaria Federico II using the Atellica IM PSA assay (Siemens Healthineers), a fully automated two-site sandwich chemiluminescent immunoassay for the quantitative determination of total PSA in human serum, according to the manufacturer’s instructions. As summarized in Table S1, the proposed assay provided PSA concentrations in good agreement with those obtained using the reference method.

**Fig. 5:**
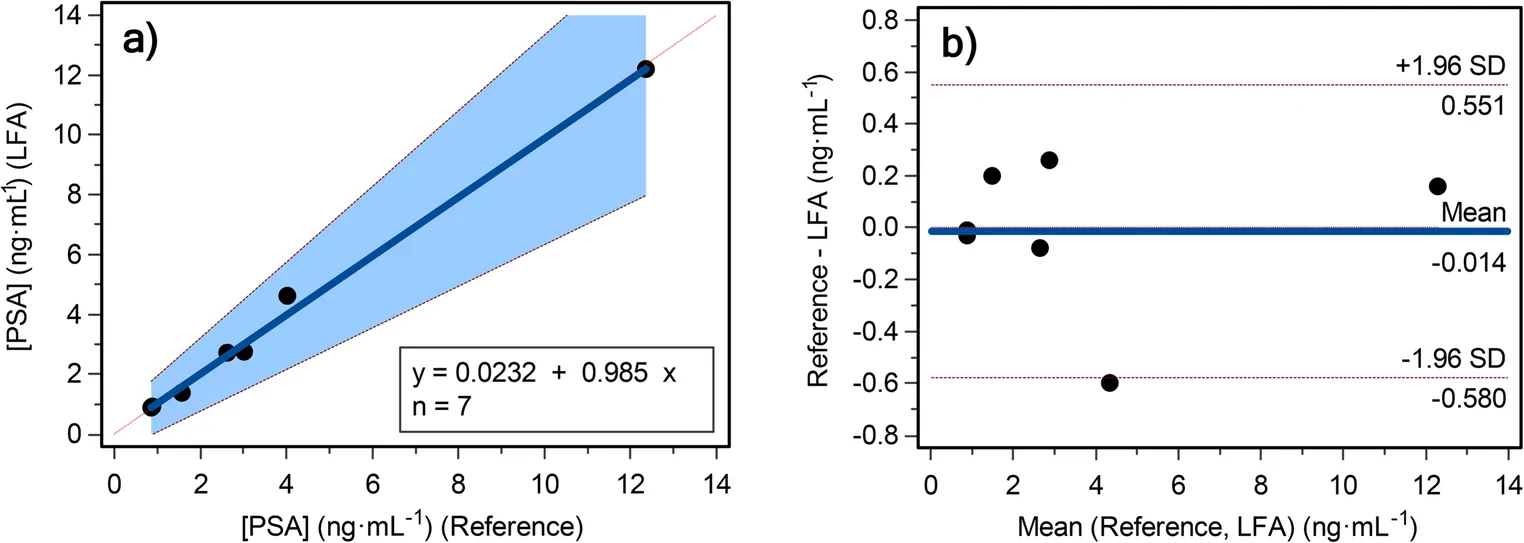
Comparison between the proposed CSMP-based LFA and the reference Atellica^®^ immunoassay for PSA determination. (**a**) Passing–Bablok regression analysis. The blue solid line represents the regression line, the red dashed line indicates the line of identity (*y* = *x*), and the light blue shaded area represents the 95% confidence interval of the regression. (**b**) Bland–Altman plot. The blue solid line represents the mean difference (bias), the brown dashed lines indicate the 95% limits of agreement, and the red dashed line denotes the zero-difference line

To explore the agreement between the CSMP-based LFA and the Atellica^®^ reference method, Passing–Bablok regression and Bland–Altman analyses were performed (Fig. 5a–b). Exploratory Passing–Bablok regression yielded an intercept of 0.023 ng·mL⁻¹, with a 95% confidence interval ranging from − 0.625 to 0.656 ng·mL⁻¹, and a slope of 0.985, with a 95% confidence interval ranging from 0.696 to 1.269. The confidence intervals included 0 and 1, respectively, and therefore did not reveal evident constant or proportional bias in the investigated samples. However, their width reflects the limited sample size, and the regression results should be regarded as preliminary.

Descriptive Bland–Altman analysis yielded a mean difference of − 0.014 ng·mL⁻¹, with limits of agreement ranging from − 0.580 to 0.551 ng·mL⁻¹. No evident concentration-dependent pattern was observed within the limited dataset.

Taken together, these analyses indicate preliminary concordance between the CSMP-based LFA and the Atellica^®^ reference method in the seven investigated samples. They should not, however, be interpreted as a formal demonstration of method equivalence or analytical interchangeability, which will require a substantially larger clinical study.

## Conclusions

This study demonstrates the use of Fe₃O₄–Au cluster–satellite magnetic particles as direct colorimetric reporters in a smartphone-quantified lateral flow assay for PSA determination in undiluted human serum. The hybrid architecture concentrates multiple plasmonic domains within a single reporter while providing an Au-rich surface for antibody immobilization. Magnetic recoverability was exploited during particle synthesis and bioconjugation to facilitate reporter isolation and the removal of unbound components, whereas signal generation during assay operation remained exclusively optical. The proposed approach therefore separates magnetic processability from analytical transduction, avoiding magnetic actuation, external excitation, and post-assay signal amplification.

Compared with other magnetic–plasmonic Au/iron oxide architectures, the present cluster–satellite particles offer a different balance between structural complexity and analytical functionality. Silica-embedded Au/iron oxide hybrids allow independent tuning of the magnetic and plasmonic components [31], while iron-based@Au core–shell structures [32] and Au–spinel ferrite heterostructures [33] provide alternative well-controlled architectures in which optical and magnetic properties can be modulated through shell formation or composition of the magnetic domain. These approaches, however, generally require multistep assembly, silica encapsulation, formation of a continuous Au shell, or high-temperature synthesis. In the present system, commercially available polymer-stabilized Fe₃O₄ clusters are directly used as magnetic scaffolds for the growth of multiple Au satellites, while magnetic recoverability facilitates purification and the Au-rich surface supports antibody immobilization and direct colorimetric transduction. A trade-off of this cluster-based design is its comparatively large hydrodynamic size, which may potentially constrain lateral-flow transport; nevertheless, efficient release and membrane migration were observed under the optimized assay conditions.

Systematic variation of CSMP extinction showed that reporter loading is a critical design parameter governing assay performance. Low particle loading produced weak optical signals, whereas excessive loading caused early saturation and compression of the concentration-dependent response. An extinction value of 4 provided the most effective balance between low-concentration detectability, optical contrast, and usable dynamic response. Under these conditions, the assay provided an estimated LoD of 0.25 ng·mL⁻¹ within a total assay time of 20 min.

The optimized assay showed limited interference from the structurally related protein hK2, recovery values within or close to the predefined 80–120% interval, and intra- and inter-assay coefficients of variation below 10% at the investigated PSA concentrations. Measurements of seven undiluted human serum samples showed preliminary agreement with the Atellica^®^ reference chemiluminescent immunoassay. Given the limited number of clinical samples, however, these results should be regarded as an initial feasibility assessment rather than a definitive validation of method agreement.

Further prospective clinical studies should evaluate larger and more heterogeneous clinical cohorts, additional potentially interfering serum components, long-term strip stability, and robustness across different smartphones, users, and acquisition conditions. The high optical contrast and smartphone-based readout may support future development toward decentralized or home-based testing; however, such applications will require dedicated usability studies, standardized image-acquisition procedures, and extensive clinical validation. Although magnetic actuation was not required in the present assay, future implementations could exploit the magnetic core for analyte preconcentration [34] or field-assisted focusing of the reporters within the lateral flow strip [35]. Overall, the results support the use of Fe₃O₄–Au cluster–satellite particles as high-payload reporters for quantitative colorimetric LFA and provide a potentially transferable design strategy for other clinically relevant biomarkers requiring rapid and minimally instrumented analysis.

## Supplementary information

Below is the link to the electronic supplementary material.

Supplementary Material 1

## Data Availability

No datasets were generated or analysed during the current study.

## Declarations

### Artificial intelligence statement

During the preparation of this manuscript, the authors used ChatGPT (OpenAI) to improve the clarity and language of the text. Figure 1 was designed by the authors in Microsoft PowerPoint and subsequently refined using ChatGPT (OpenAI) to enhance its graphical appearance (e.g., rendering of materials and colors). All AI-assisted outputs were critically reviewed, edited, and scientifically validated by the authors, who take full responsibility for the final content of the manuscript.
