# Supplementary material for: Fe3O4–Au cluster–satellite particles as direct colorimetric reporters in a smartphone-quantified lateral flow immunoassay for prostate-specific antigen
Source: Mikrochim Acta. 2026 Sep 25;193(10):696. doi: 10.1007/s00604-026-08422-8 (PMC13615018; doi:10.1007/s00604-026-08422-8)
Supplement: Supplementary file 1 [file 604_2026_8422_MOESM1_ESM.pdf]

Supplementary Information

Fe_3O_4–Au Cluster–Satellite Particles as Direct Colorimetric Reporters in a Smartphone-Quantified Lateral Flow Immunoassay for Prostate-Specific Antigen

Daniele Marra[1], Erica Cavaliere[1], Daniela Terracciano[2], Luca Livio Levorato[1], Tina D'Aponte[1], Vincenzo Iannotti[1], Francesco Gentile[3], Raffaele Velotta[1*], Bartolomeo Della Ventura[1*]

[1]Department of Physics "Ettore Pancini", University of Naples Federico II, Via Cintia 26, 80126, Naples, Italy

[2]Department of Translational Medical Sciences, University of Naples Federico II, Via S.Pansini, 5, 80131, Naples, Italy

[3]Department of Experimental and Clinical Medicine, University Magna Græcia, Campus Universitario "Salvatore Venuta", Viale Europa - 88100 Catanzaro, Italy.

*Corresponding authors: Raffaele Velotta (e-mail: rvelotta@unina.it), Bartolomeo Della Ventura (e-mail: bartolomeo.dellaventura@unina.it)

Table of Contents

Figure S1. Schematic representation of the antibody conjugation procedure using UV activation and microfluidic immobilization onto CSMPs.

Figure S2. Normalized extinction spectra of CSMPs before functionalization, after antibody conjugation, and after BSA blocking.

Figure S3. Custom 3D-printed smartphone holder for standardized LFA image acquisition.

Figure S4. Calibration curve used for grayscale calibration of LFA strip images.

Figure S5. Individual test- and control-zone signals used for calculation of the T/C ratio.

Table S1. Comparison of PSA measurements in human serum samples

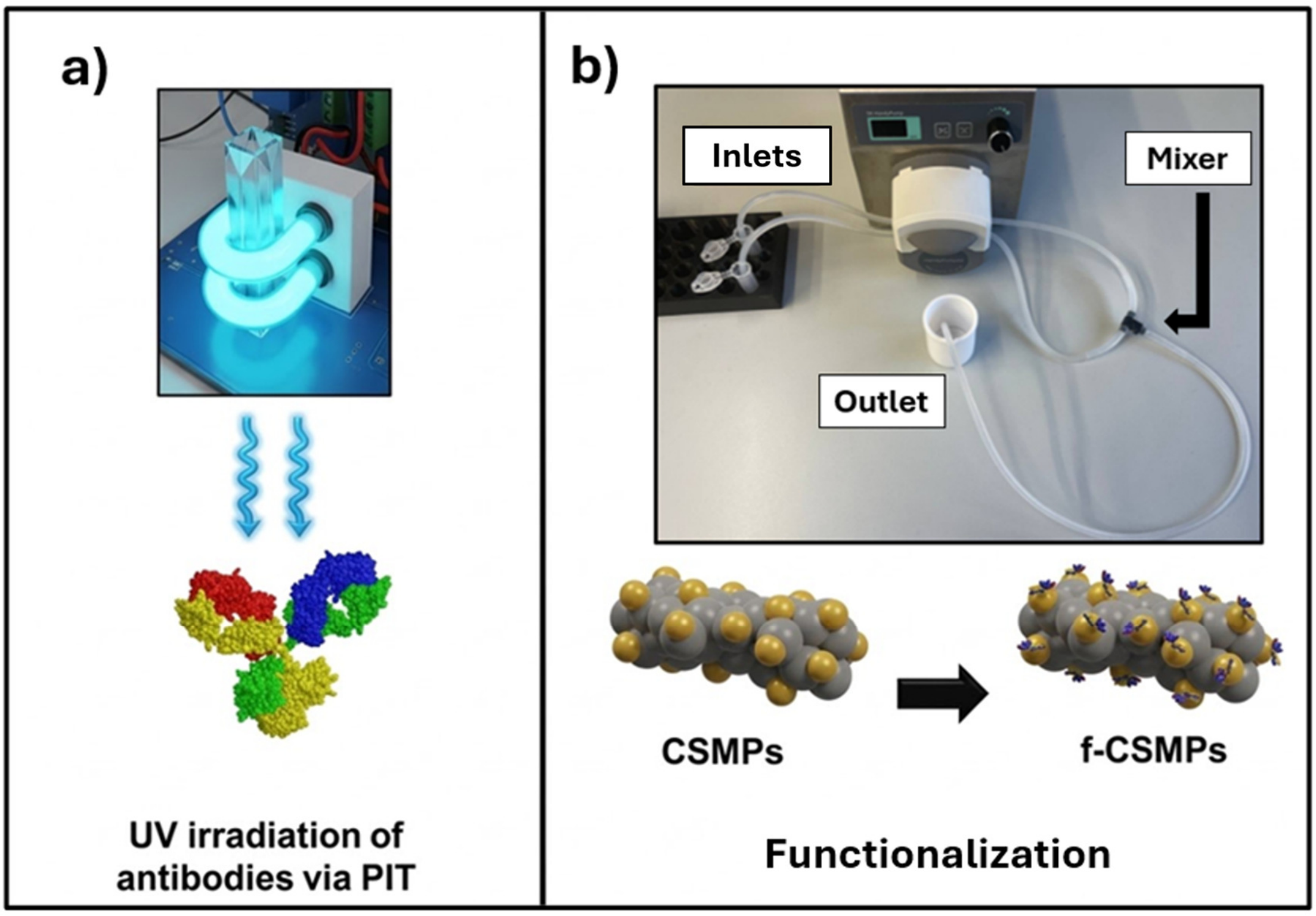

Figure S1. Sequence of steps involved in the antibody conjugation procedure: (a) antibodies are activated by UV irradiation using Trylight®, and (b) the activated antibodies are mixed with a CSMPs suspension within a microfluidic system, where efficient mixing promotes their immobilization onto the CSMPs surface, resulting in the formation of functionalized CSMPs.

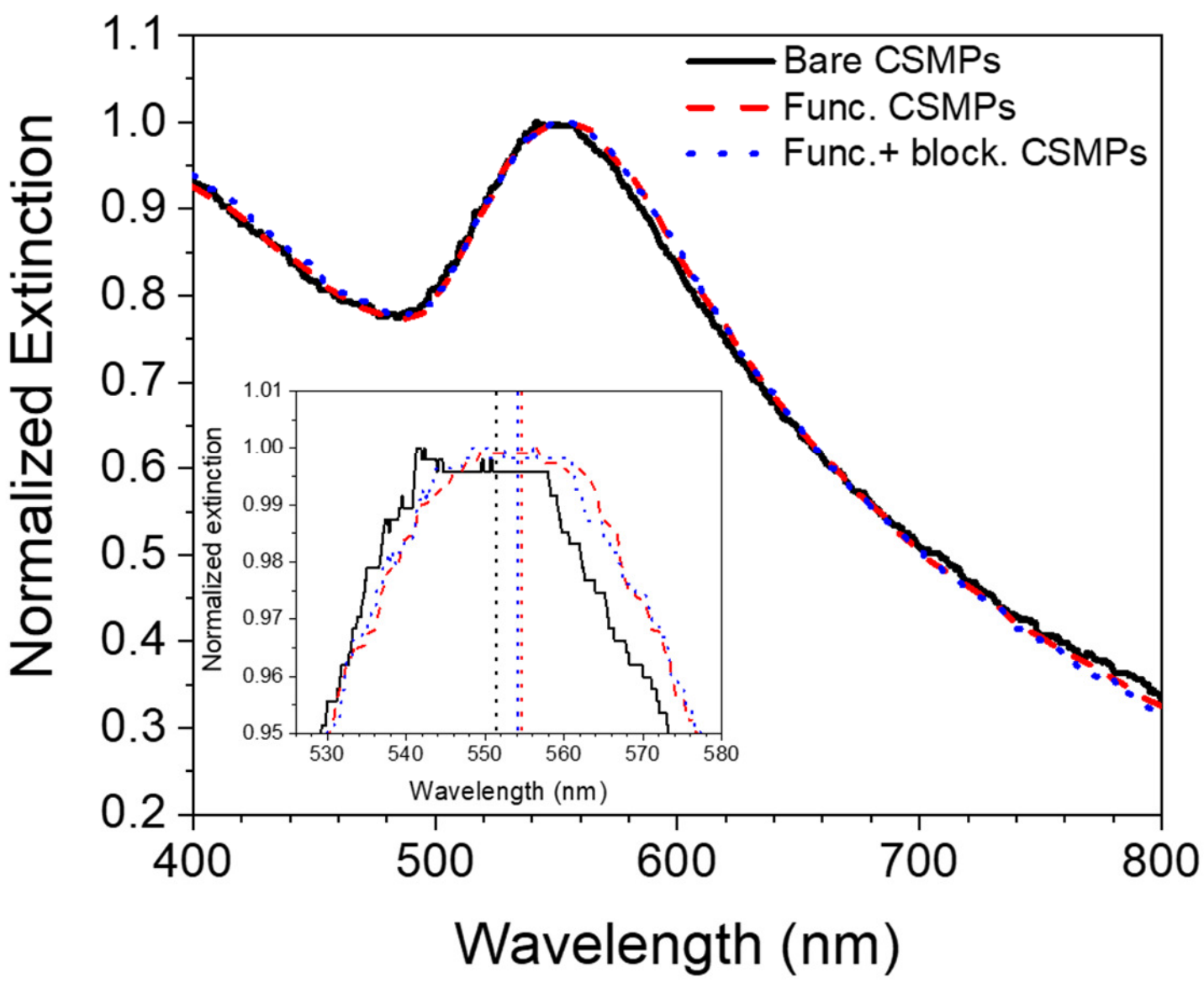

Figure S2. Normalized extinction spectra of CSMPs before (black solid line), after antibody functionalization (red dashed line), and after BSA blocking (blue dotted line). The inset shows a magnified view in the 526–580 nm range, highlighting a ~3 nm red shift following functionalization. The observed shift of the plasmonic peak confirms the successful immobilization of anti-PSA antibodies on the particle surface.

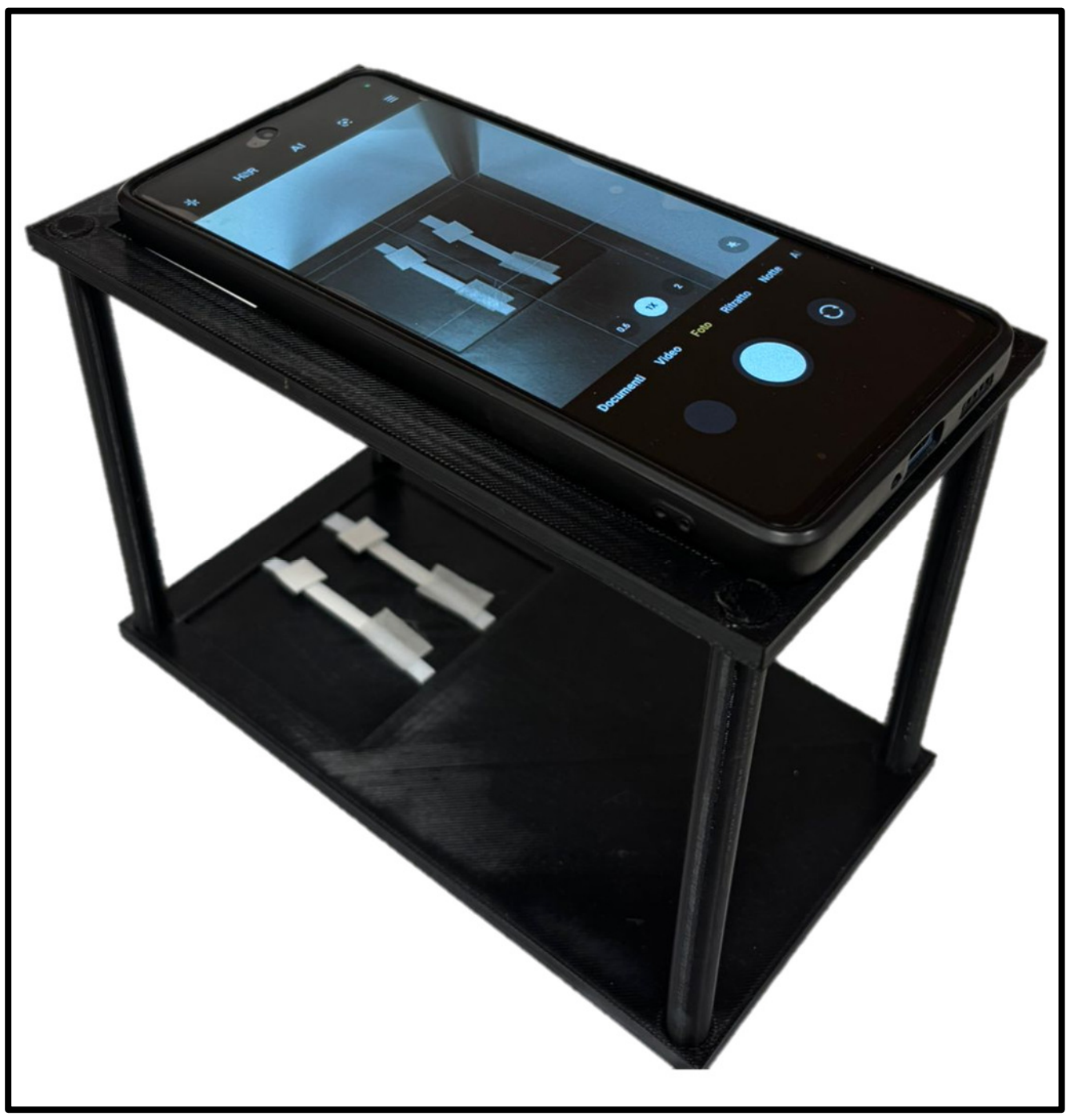

Figure S3. Custom 3D-printed smartphone holder used to ensure reproducible imaging conditions during LFA analysis. The device consists of an upper platform supporting the smartphone at a fixed height of 12 cm and a lower compartment designed to accommodate the LFA strips. This configuration ensures a constant imaging geometry and was maintained in the same position for all acquisitions to guarantee consistent photographic conditions.

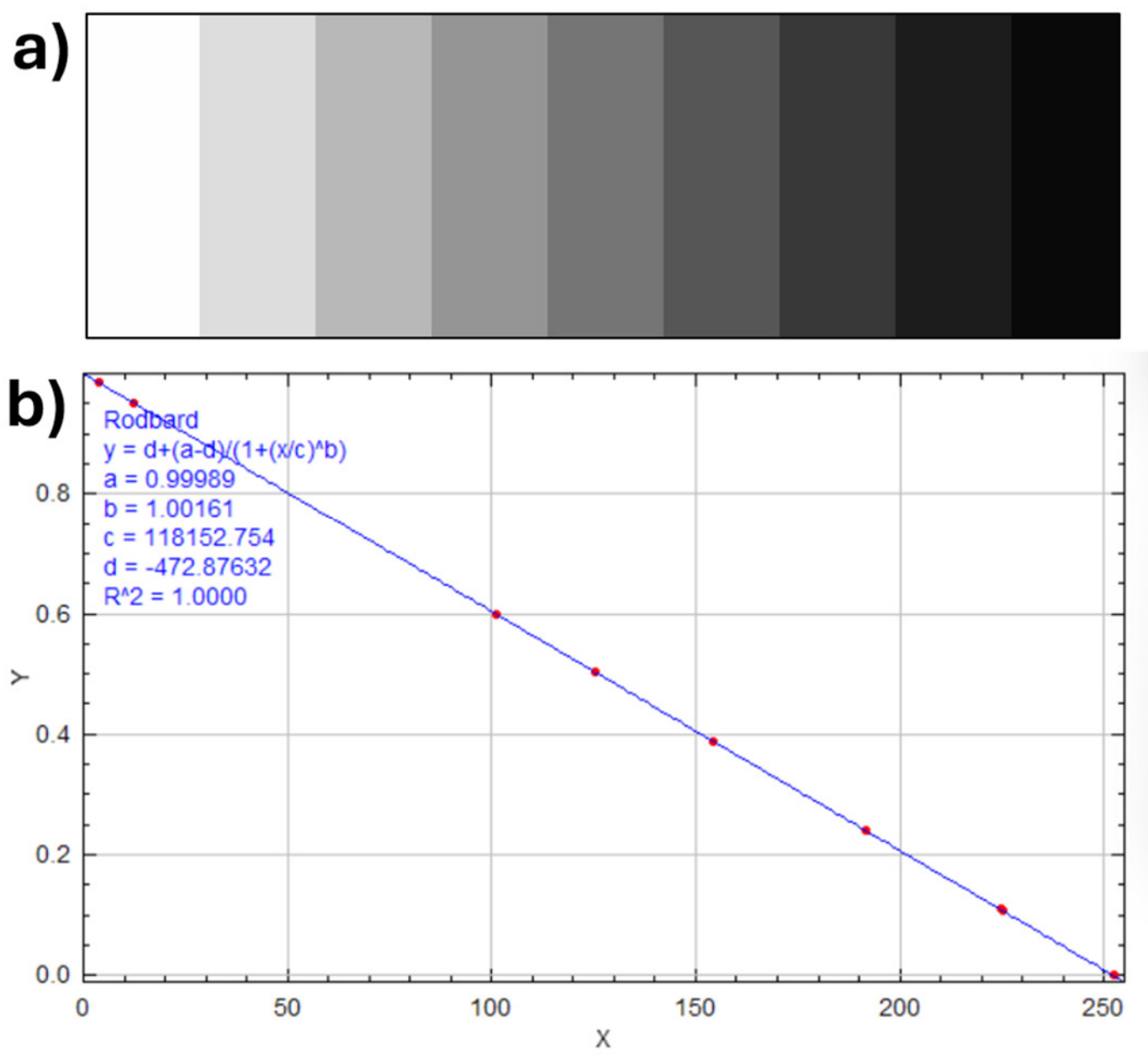

Figure S4. (a) Grayscale calibration card used to calibrate image intensity prior to analysis. **(b)** Calibration curve generated from the normalized grayscale values (black = 1, white = 0) and applied to LFA image analysis.

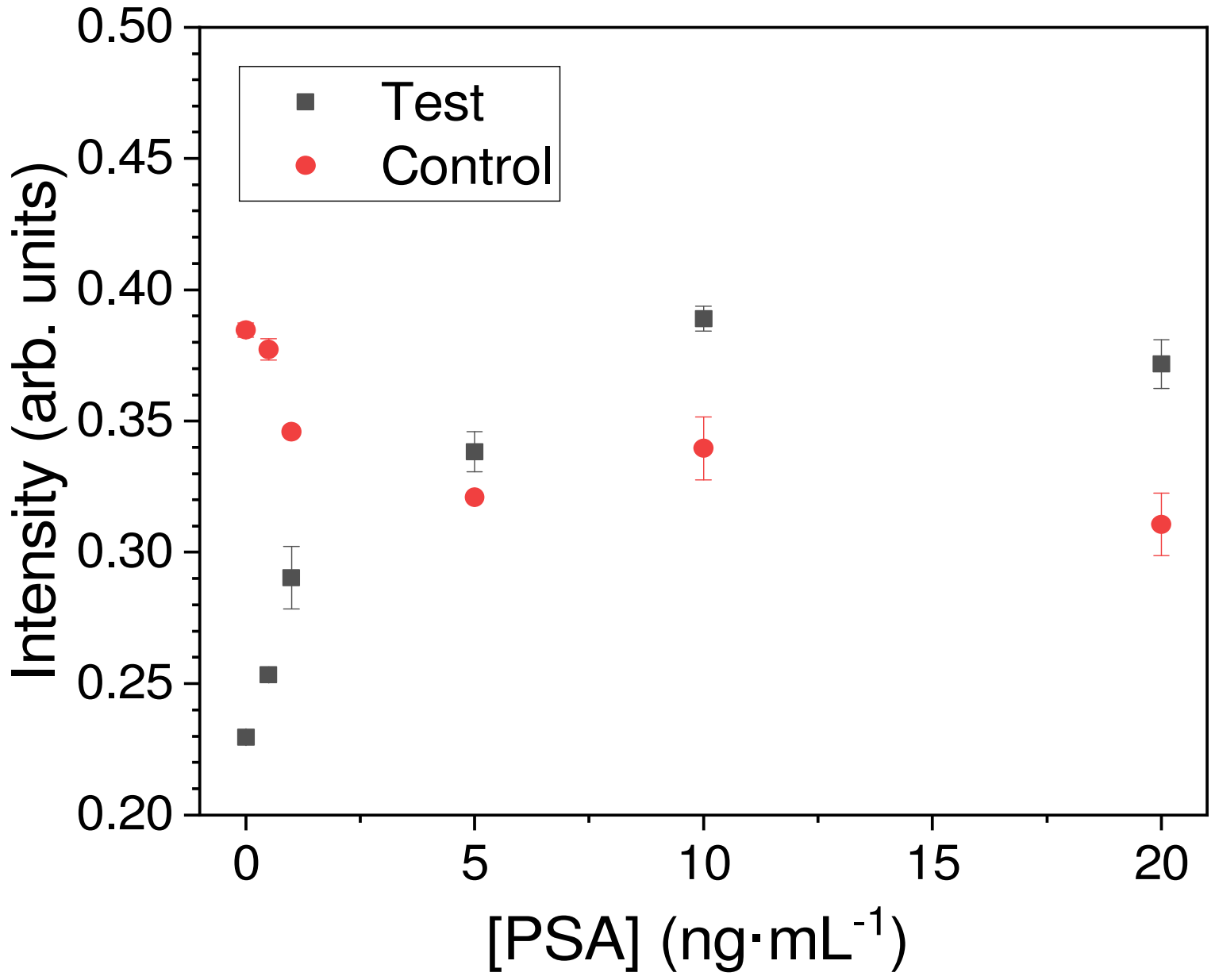

Figure S5. Mean test- and control-zone intensities measured for the optimized CSMP reporter loading (Ext 4) at PSA concentrations ranging from 0 to 20 ng·mL^{-1} in spiked human serum. Test and control intensities were measured on the same strip, and the T/C ratio was calculated separately for each replicate before averaging. Data are reported as mean ± standard deviation of triplicate measurements.

Atellica® IM PSA assay ($ng·mL^{-1}$)	CSMPs Ext 4 [PSA] ($ng·mL^{-1}$)	CSMP/Atellica® ratio
4.03	4.63 ± 0.07	115 ± 2
3.01	2.75 ± 0.25	91 ± 8
2.62	2.7 ± 0.15	103 ± 6
12.36	12.2 ± 0.78	99 ± 6
1.58	1.38 ± 0.08	87 ± 5
0.86	0.89 ± 0.06	104 ± 7
0.89	0.9 ± 0.06	101 ± 7

Table S1. Comparison of PSA concentrations measured in human serum samples using the reference Atellica® IM PSA chemiluminescent immunoassay and the proposed CSMP-based lateral flow immunoassay. CSMP-based LFA results are reported as mean ± standard deviation of triplicate measurements. The CSMP/Atellica ratio was calculated as the concentration measured by the proposed assay divided by that obtained using the reference method and is reported as a percentage.
